# Taxonomic and functional profiles of *Coffea canephora* endophytic microbiome in the Central Highlands region, Vietnam, revealed by analysis of 16S rRNA metagenomics sequence data

**DOI:** 10.1016/j.dib.2022.108372

**Published:** 2022-06-10

**Authors:** Dinh Minh Tran

**Affiliations:** Institute of Biotechnology and Environment, Tay Nguyen University, Buon Ma Thuot, Dak Lak 630000, Vietnam

**Keywords:** Endophytic microbiome dataset, *Coffea canephora* L., metagenomic next-generation sequencing, The Central Highlands

## Abstract

Vietnam is the largest producer of Robusta coffee (*Coffea canephora* L.) [1]. Among regions in Vietnam, the Central Highlands is the capital of the coffee plantation and production [2]. Previous works have established a dataset of rhizospheric microbial diversity and its functionality to develop sustainable Robusta coffee production techniques in this region [3], [4], [5], [6]; however, a dataset of the endophytic microbiome of this plant species has been found is still unknown. The work presented here is the first report on the microbial and functional diversity of the endophytic microbiome of *Coffea canephora* L. grown in the region. A representative root sample was obtained by mixing five different root samples collected from a 6-year-old Robusta coffee field in Dak Lak Province, the Central Highlands, on 30 October 2021. After that, 16S rRNA metagenomic next-generation sequencing was conducted on the sample using the Illumina MiSeq platform. The raw sequence of endophytic microbiome data in this work was uploaded in Fastq format on NCBI with Bioproject PRJNA821717 and can be accessed at https://www.ncbi.nlm.nih.gov/Traces/study/?acc=PRJNA821717. The dataset can be useful for understanding basic information about the prokaryotic ecology of this important plant in the Central Highlands, Vietnam. The data can also be valuable for developing cultivation techniques for sustainable *Coffea canephora* L. production by applying indigenous microbial resources.

## Specifications Table

**Table:** 

Subject	Microbiology: *Microbiome*
Specific subject area	Metagenomics, Molecular biology, Bioinformatics
Type of data	Figures, Fastq files
How the data were acquired	Metagenomic sequencing with V1-V9 regions of the 16S rRNA genes using Illumina MiSeq platform
Data format	Raw and Analyzed
Description of data collection	Microbial metagenomic DNA extraction was performed using the DNeasy PowerSoil kit (Qiagen, Germany). As Tran et al. described previously, the V1–V9 regions of the 16S rRNA genes were amplified [6], [7], [8]. Libraries of 16S rRNA gene amplicons were prepared using the Swift amplicon 16S plus ITS panel kit (Swift Biosciences, USA). Finally, the Illumina MiSeq platform (2 × 150 bp paired ends) was used to perform the library 16S rRNA gene amplicon sequencing
Data source location	Ward/City/Province: Khanh Xuan/Buon Ma Thuot/Dak Lak Region: The Central Highlands Country: Vietnam Latitude and longitude coordinates for collected samples: 12°38′55.20′′N,107°59′22.06′′E
Data accessibility	Data are available at the NCBI with Bioproject PRJNA821717 (https://www.ncbi.nlm.nih.gov/Traces/study/?acc=PRJNA821717)

## Value of the Data

• Data provide information on endophytic microbiome profiles of *Coffea canephora* L. and its functionality cultivated in the Central Highlands, Vietnam.
• Data provide information on the difference between rhizospheric and endophytic microbiome profiles and their functionality of *Coffea canephora* L. cultivated in the Central Highlands, Vietnam.
• Data can be valuable for comparing endophytic microbiome profiles of Robusta coffee cultivated in the Central Highlands, Vietnam, and other regions.
• Data can be valuable for further study on the conservation and utilization of endophytic microbial resources for sustainable Robusta coffee production in the Central Highlands by using indigenous microorganisms.

## 1 Data Description

### 1.1 Taxonomic analysis of endophytic microbiome in the root of Robusta coffee

**Fig. 1:**
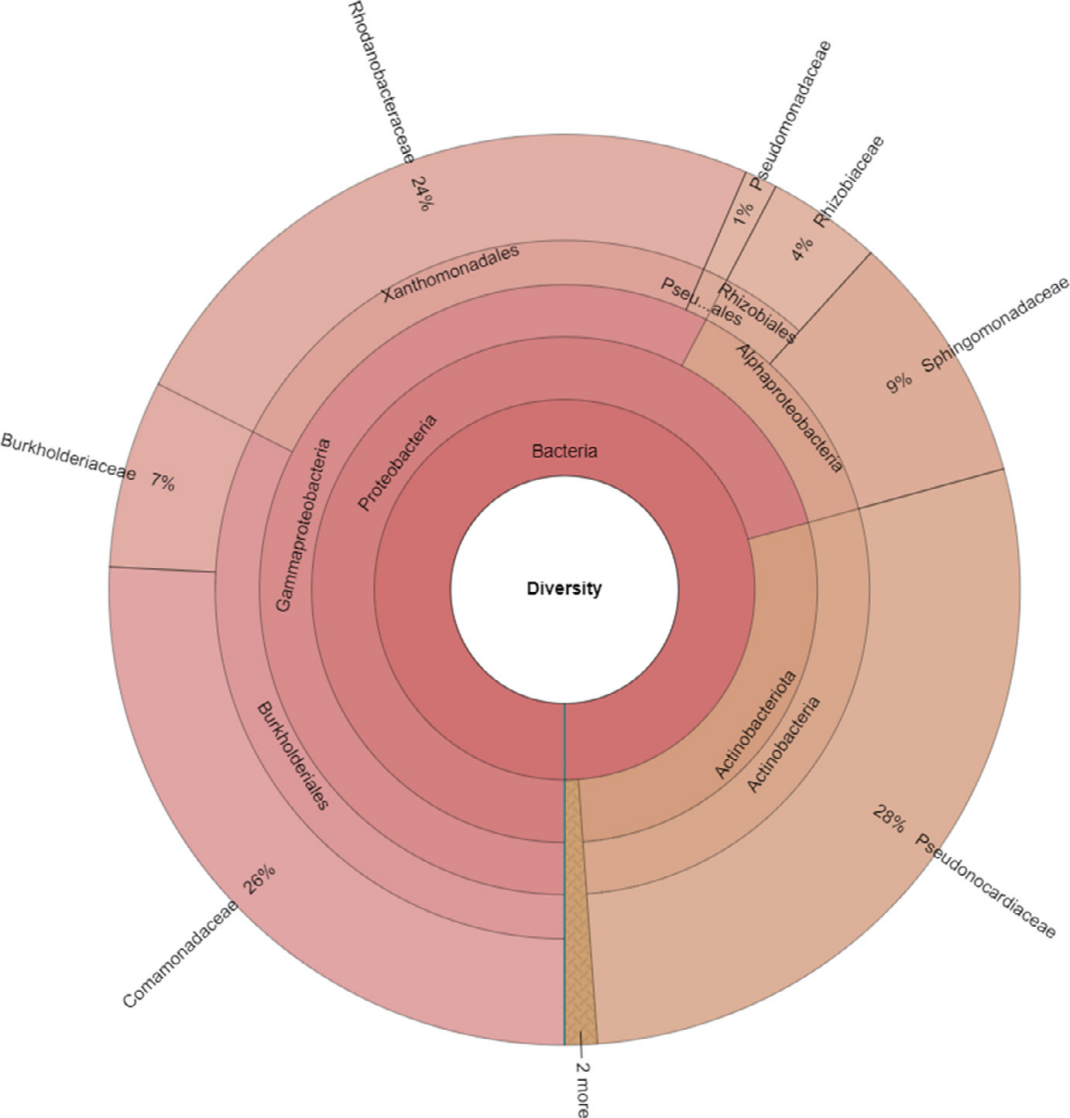
Taxonomic profiles of the endophytic microbiome of *Coffea canephora* in the Central Highlands region, Vietnam.

The results showed that a total of 174,988 reads were identified. Taxonomic analysis (Fig. 1) showed four phyla from the sample were identified. Among them, Proteobacteria (70.72%) were found to be the most abundant, followed by Actinobacteriota (28.12%), Acidobacteriota (0.58%), and Bacteroidota (0.58%). Furthermore, five classes were identified in which, Gammaproteobacteria were 57.68%, Actinobacteria were 28.12%, Alphaproteobacteria were 13.04%, Holophagae were 0.58%, and Bacteroidia were 0.58%. Of the eight bacterial orders detected, Burkholderiales (32.24%) were shown to be the most dominant, followed by Pseudonocardiales (28.12%), Xanthomonadales (24.06%), Sphingomonadales (8.99%), Rhizobiales (4.06%), Pseudomonadales (1.16%), Holophagales (0.58%), and Chitinophagales (0.58%). Moreover, nine families were detected; among them, Pseudonocardiaceae (28.12%) were found to be the most abundant, followed by Comamonadaceae (25.8%), Rhodanobacteraceae (24.06%), Sphingomonadaceae (8.99%), Burkholderiaceae (6.67%), Rhizobiaceae (4.06%), Pseudomonadaceae (1.16%), Holophagaceae (0.58%), and Chitinophagaceae (0.58%). Finally, 210 out of 345 genera were identified from the root sample of Robusta coffee; among the identified genera, the genus Amycolatopsis (41.43%) was the most popular, followed by Sphingobium (14.76%) and Dyella (12.38%).

**Fig. 2:**
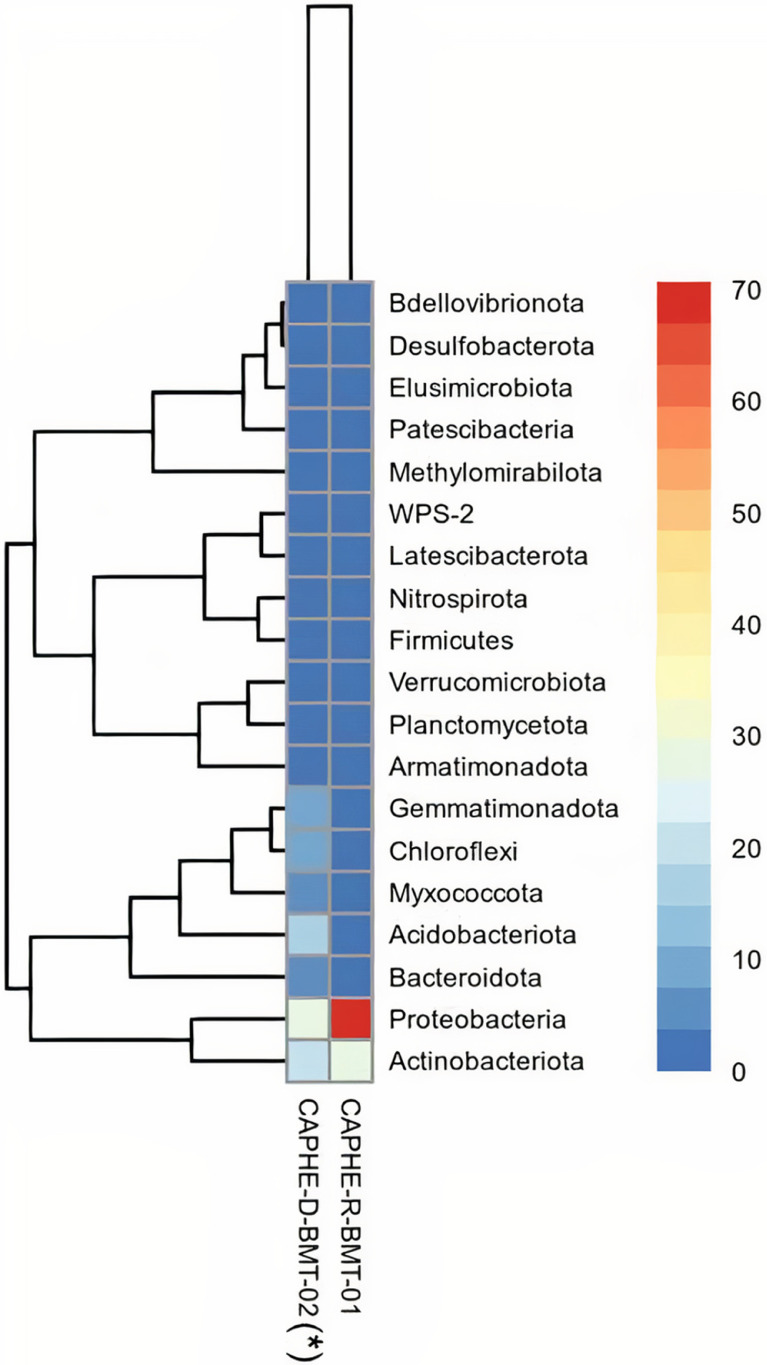
Taxonomic profiles of the endophytic and rhizospheric microbiome in the root of *Coffea canephora* in the Central Highlands region, Vietnam.

Note: CAPHE-R-BMT-01, data from this study; CAPHE-D-BMT-02(*), data from Tran et al. [6].

A comparison of taxonomic profiles of the endophytic and rhizospheric microbiome in the root of Robusta coffee (Fig. 2) showed that there were four phyla, Proteobacteria, Actinobacteriota, Acidobacteriota, and Bacteroidota, present in the root of Robusta coffee, among them bacteria belonging to Proteobacteria and Actinobacteriota were the most popular; however, those were 28 in the rhizosphere of the plant and Proteobacteria, Actinobacteriota, Acidobacteriota, Gemmatimonadota, Chloroflexi, and Myxococcota were the most abundant.

### 1.2 Functional analysis of endophytic microbiome in the root of Robusta coffee

**Fig. 3:**
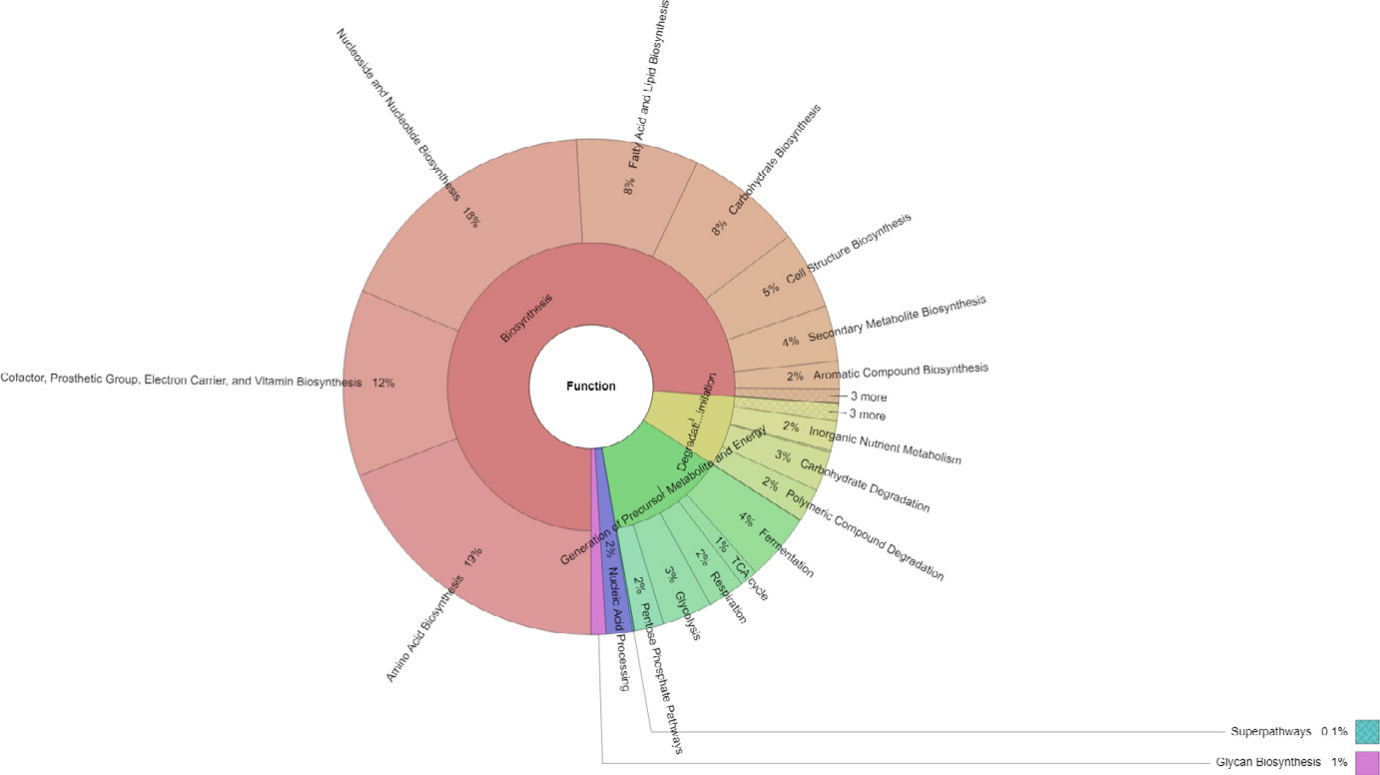
Functional profiles of the endophytic microbiome of *Coffea canephora* in the Central Highlands region, Vietnam.

**Fig. 4:**
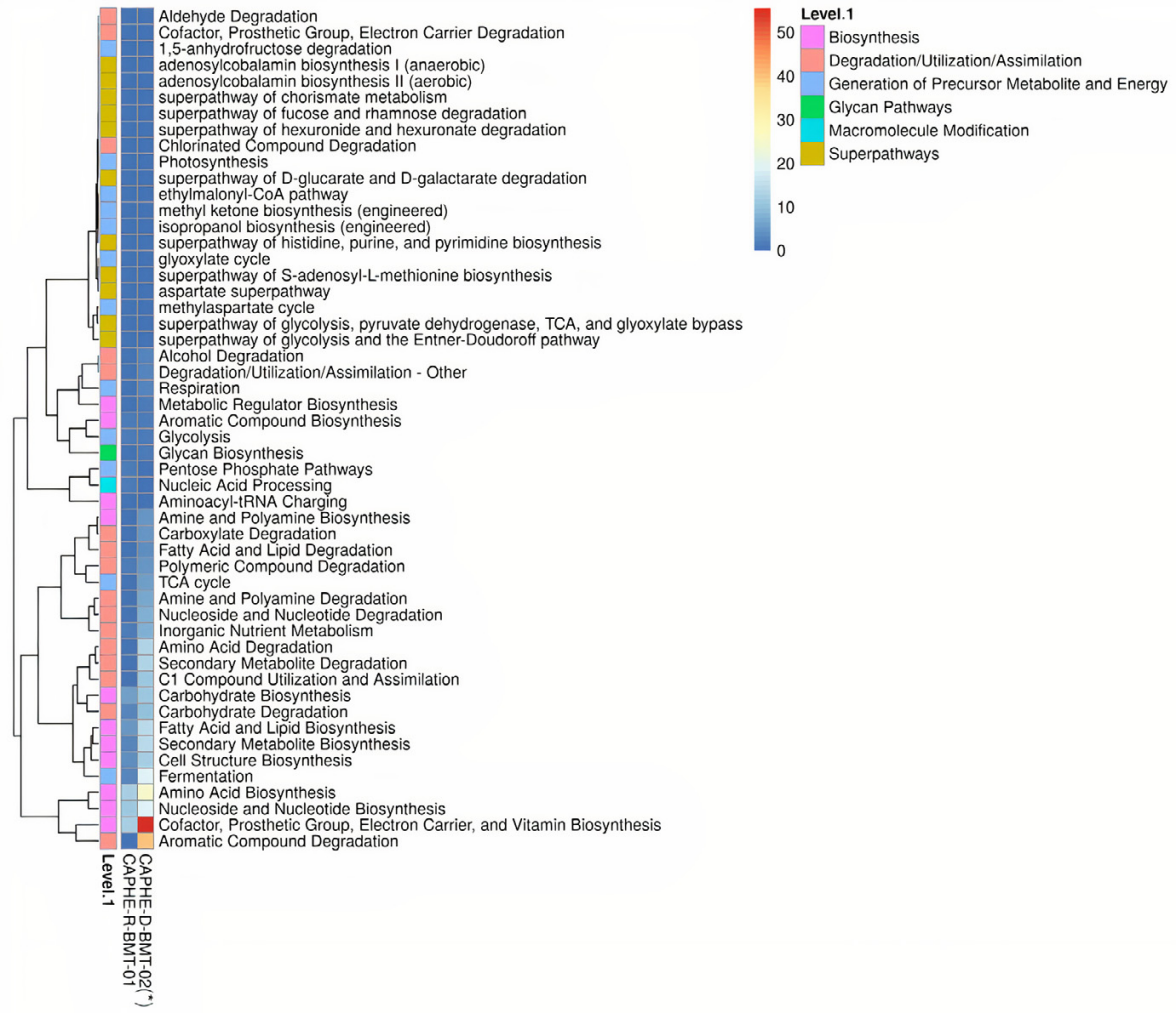
Functional profiles of the endophytic and rhizospheric microbiome in the root of *Coffea canephora* in the Central Highlands region, Vietnam.

Note: CAPHE-R-BMT-01, data from this study; CAPHE-D-BMT-02(*), data from Tran et al. [6].

As a result, shown in Fig. 3, the primary function of the endophytic microbiome of Robusta coffee was biosynthesis (69.67%), followed by the generation of precursor metabolites and energy (13.15%), and degradation/utilization/assimilation (7.98%). Among the functions involved in biosynthesis, amino acid biosynthesis (19.16%) was the most abundant, followed by nucleoside and nucleotide biosynthesis (17.73%); cofactor, prosthetic group, electron carrier, and vitamin biosynthesis (12.15%); fatty acid and lipid biosynthesis (7.97%); carbohydrate biosynthesis (7.61%); cell structure biosynthesis (5.09%); and secondary metabolite biosynthesis (3.62%). Moreover, the result in Fig. 4 showed that amino acid biosynthesis; nucleoside and nucleotide biosynthesis; cofactor, prosthetic group, electron carrier; and vitamin biosynthesis were the most abundant functions endophytic microbiome of Robusta coffee. However; cofactor, prosthetic group, electron carrier, and vitamin biosynthesis; aromatic compound degradation; amino acid biosynthesis; fermentation, cell structure biosynthesis, nucleoside and nucleotide biosynthesis; fatty acid and lipid biosynthesis; secondary metabolite biosynthesis; carbohydrate degradation; carbohydrate biosynthesis; C1 compound utilization and assimilation; secondary metabolite degradation; and amino acid degradation was the primary functions of the rhizospheric microbiome of the coffee.

## 2 Experimental Design, Materials and Methods

### 2.1 Rhizospheric soil sampling

Five root samples (approximately 50 g each, 5–30 cm in depth) of *Coffea canephora* L. were collected from five different positions of a 6-year-old coffee field in Khanh Xuan Ward, Buon Ma Thuot City, Dak Lak Province, on October 30, 2021. The collection was the same positions, tree, and time as the rhizospheric soil sampling in the previous study [6]. After that, root samples were mixed and combined into one representative sample. The sample was stored under cool conditions (4°C) while transferred to the laboratory and kept at −80°C until metagenomic DNA extraction.

### 2.2 Isolation of microbial genomic DNA

Metagenomic DNA was extracted from 250 mg of the root sample using the DNeasy PowerSoil kit (Qiagen, Germany) based on the manufacturer's instructions.

### 2.3 Library preparation and 16S rRNA metagenomic sequencing

Library preparation and 16S rRNA metagenomic sequencing were conducted as Tran et al. [6], [7], [8] described. Briefly, the V1–V9 regions of the 16S rRNA genes were amplified, and the Swift amplicon 16S plus internal transcribed spacer panel kit (Swift Biosciences, USA) was used to prepare libraries of 16S rRNA gene amplicons. Lastly, the Illumina MiSeq platform (2 × 150 bp paired ends) was used to sequence the 16S rRNA gene amplicon from the library.

### 2.4 Taxonomic and functional analyses

Taxonomic and functional profiles of endophytic prokaryotes in the root sample were analyzed using the method described by Tran et al. [6], [7], [8]. Briefly, bcl2fastq was used to demultiplex raw basecall files. Trimmomatic (version 0.39) and Cutadapt (version 2.10) were used to remove adapters, primers, and low-quality sequences (average score of <20 and read length of <100 bp). The q2-dada2 plugin and QIIME2 pipeline (version 2020.8) were used for clustering and dereplicating reads into amplicon sequence variants. QIIME2 aligned with the SILVA SSURef reference database (version 138) was used for taxonomic analysis. Finally, PICRUSt2 (version 2.3.0-b) and MetaCyc databases were used to predict the functional profiles of endophytic prokaryotes based on the metagenomic sequences.

## Ethics Statements

None.

## CRediT Author Statement

**Dinh Minh Tran:** Conceptualization, Methodology, Investigation, Formal analysis, Software, Data curation, Validation, Visualization, Writing – original draft, Writing – review & editing.

## Declaration of Competing Interest

The authors declare that they have no known competing financial interests or personal relationships that could have appeared to influence the work reported in this paper.

## Data Availability

Taxonomic and functional profiles of *Coffea canephora* endophytic microbiome in the Central Highlands region, Vietnam, revealed by analysis of 16S rRNA metagenomics sequence data (Original data).
